# Neck circumference as a new anthropometric indicator for prediction of insulin resistance and components of metabolic syndrome in adolescents: Brazilian Metabolic Syndrome Study

**DOI:** 10.1590/0103-0582201432210713

**Published:** 2014-06

**Authors:** Cleliani de Cassia da Silva, Mariana Porto Zambon, Ana Carolina J. Vasques, Ana Maria de B. Rodrigues, Daniella Fernandes Camilo, Maria Ângela R. de G. M. Antonio, Roberta Soares L. Cassani, Bruno Geloneze

**Affiliations:** 1 Faculdade de Ciências Médicas da Unicamp; Laboratório de Investigação em Metabolismo e Diabetes (Limed) - Gatrocentro-Unicamp, Campinas, SP, Brasil

**Keywords:** insulin resistance, metabolic syndrome x, adolescent, adiposity

## Abstract

**OBJECTIVE:**

To evaluate the correlation between neck circumference and insulin resistance and components of metabolic syndrome in adolescents with different adiposity levels and pubertal stages, as well as to determine the usefulness of neck circumference to predict insulin resistance in adolescents.

**METHODS:**

Cross-sectional study with 388 adolescents of both genders from ten to 19 years old. The adolescents underwent anthropometric and body composition assessment, including neck and waist circumferences, and biochemical evaluation. The pubertal stage was obtained by self-assessment, and the blood pressure, by auscultation. Insulin resistance was evaluated by the Homeostasis Model Assessment-Insulin Resistance. The correlation between two variables was evaluated by partial correlation coefficient adjusted for the percentage of body fat and pubertal stage. The performance of neck circumference to identify insulin resistance was tested by Receiver Operating Characteristic Curve.

**RESULTS:**

After the adjustment for percentage body fat and pubertal stage, neck circumference correlated with waist circumference, blood pressure, triglycerides and markers of insulin resistance in both genders.

**CONCLUSIONS:**

The results showed that the neck circumference is a useful tool for the detection of insulin resistance and changes in the indicators of metabolic syndrome in adolescents. The easiness of application and low cost of this measure may allow its use in Public Health services.

## Abstract (es)

**OBJETIVO:**

Evaluar la correlación de la circunferencia del cuello con resistencia a la insulina y con los componentes del síndrome metabólico en adolescentes con distintos niveles de adiposidad y estadios puberales, así como determinar la utilidad de la circunferencia del cuello como un parámetro en la predicción de resistencia a la insulina en adolescentes.

**MÉTODOS:**

Estudio transversal en el que se evaluó a 388 adolescentes de ambos sexos, de los 10 a los 19 años. Los adolescentes fueron sometidos a evaluación antropométrica y de composición corporal, incluyendo circunferencias del cuello y de la cintura, y la evaluación bioquímica. El estadio puberal se obtuvo mediante autoevaluación y la presión arterial, por el método de ausculta. Se evaluó la resistencia a la insulina por el Homeostasis Model Assessment-Insulin Resistence. La correlación entre dos variables fue evaluada con el coeficiente de corrrelación parcial ajustado para el porcentaje de grasa corporal y el estadio puberal. El desempeño de la circunferencia del cuello para identificar resistencia a la insulina fue evaluado por la Receiver Operating Characteristic Curve y se consideró p<0,05.

**RESULTADOS:**

Después del ajuste para el porcentaje de grasa corporal y estadio puberal, la circunferencia del cuello se correlacionó con la circunferencia de la cintura, presión arterial, triglicéridos y marcadores de resistencia a la insulina en ambos sexos.

**CONCLUSIONES:**

Los resultados demostraron que la circunferencia del cuello es una herramienta útil para detectar la resistencia a la insulina y la alteración en los indicadores de síndrome metabólico en adolescentes. La facilidad de aplicación y el bajo costo pueden hacer su uso viable en servicios de Salud Pública.

## Introduction

Body composition and body fat distribution are associated with complications such as insulin resistance (IR), dyslipidemia, diabetes mellitus type 2, and cardiovascular diseases (CVD) in adults, children, and adolescents^(^ 1 ^,^ 2 ^)^. Imaging studies - computed tomography, magnetic resonance and dual energy X-ray absorptiometry (DEXA) - are the gold standard tools for evaluating body adiposity, but they are not applicable in all situations and have a high cost. In epidemiological studies and in clinical practice, anthropometric measurements such as body mass index (BMI), waist circumference (WC), and neck circumference (NC) are valued for being quicker, non-invasive, more accesible, and cheaper than imaging studies, making them easier to apply^(^ 3 ^)^.

Having international reference standards, BMI is currently the most common measurement for the diagnosis of overweight and obesity in children and adolescents. However, it cannot always assess people's individual risks of endocrine and metabolic complications, since it does not evaluate body fat distribution^(^ 3 ^)^. WC has been used in the diagnosis of metabolic syndrome (MS), as a predictor of IR, and in the assessment of risk factors for CVD in adolescents^(^ 4 ^)^. However, there are limitations for this age group, such as the lack of a standard method of measurement^(^ 5 ^-^ 7 ^)^; lack of an international standard due to ethnic variation; lack of a cutoff point for cardiovascular and metabolic risks; and practical obstacles, such as the need to undress, particularly during winter. This may also have a psychological effect on this age group^(^ 8 ^)^.

Studies involving adults have suggested using NC as an alternative to WC, since NC is a simpler and more practical anthropometric indicator, not affected by postprandial abdominal distension or by respiratory movements. According to these studies, this measurement provides consistent results for excess subcutaneous fat in the upper body^(^ 8 ^-^ 10 ^)^. An increase in NC, as well as visceral fat, is associated with cardiometabolic risks^(^ 10 ^)^. Studies with adults also found a positive correlation of NC with IR, components of MS, and cardiovascular risk factors^(^ 8 ^-^ 10 ^)^. Studies that evaluate NC in adolescents are rare.

Therefore, considering that NC can be easily obtained in epidemiological studies and in clinical practice and that early identification of metabolic alterations helps prevent diseases, this study aimed to: 1) evaluate the correlation of NC with IR and components of MS in adolescents with different body fat levels and at different puberty stages; and 2) identify whether NC may be a predictor of IR in this age range.

## Method

This cross-sectional study is based on a larger study named *Brazilian Metabolic Syndrome Study *(BRAMS), which evaluates clinical, anthropometric, metabolic, and hormonal aspects of insulin resistance syndrome in children and adolescents.

Three hundred and eighty-eight adolescents of both sexes, between ten and 19 years old, were evaluated. A convenience sampling strategy was used. The sample comprised adolescents treated at an outpatient clinic for obese children and adolescents in the Hospital de Clínicas da Universidade Estadual de Campinas, at primary health care units, adolescents from public schools, and adolescents from institutions who offer socio-educational programs for this age range. The study included overweight, obese, and normal weight subjects, according to criteria from the *Centers for Disease Control and Prevention* (CDC)^(^ 11 ^)^. Exclusion criteria were: cervical lymph nodes or deformities, goitre, late neuropsychological and motor development, congenital syndromes, hepatopathy, nephropathy, metabolic disorders (such as type 1 diabetes, hypothyroidism, and hyperthyroidism) and use of systemic corticosteroids.

Weight and height were measured according to the techniques proposed by Gordon et al^(^ 12 ^)^. From these measurements, the z score for body mass index-for-age (BMIz) was determined. WC was measured with a measuring tape at the midpoint between the iliac crest and the last rib^(^ 13 ^)^. NC was measured at the midpoint of the neck^(^ 9 ^)^. Body fat percentage (BF%) was assessed by tetrapolar bioelectrical impedance^(^ 14 ^,^ 15 ^)^ using the BIA 310 Bioimpedance Analyzer device, as proposed by Lukaski et al^(^ 14 ^)^. Blood pressure was measured by the auscultatory method using a mercury column sphygmomanometer, as recommended by the Brazilian Society of Cardiology^(^ 16 ^)^. Sexual maturation was self-assessed following the criteria suggested by Tanner^(^ 17 ^)^. For the classification between prepubertal (1-2) and pubertal (3-5), we considered breasts in females and external genitalia in males.

For biochemical evaluation, blood samples were collected after an overnight 12-hour fast. Plasma glucose, total cholesterol (TC), HDL, triglycerides (TG), and gamma-glutamyl transferase (GGT) were determined using the enzymatic colorimetric method, and the LDL-cholesterol fraction was calculated with the Friedewald equation^(^ 18 ^)^. Plasma uric acid was measured by the uricase method; glycated hemoglobin (HbA1C), by high-performance liquid chromatography (HPLC); serum concentrations of aspartate aminotransferase (AST) and alanine aminotransferase (ALT), by the UV-kinetic method; and plasma insulin, by chemiluminescence. IR was assessed by the *Homeostasis Model Assessment-Insulin Resistance* (HOMA1-IR) index, calculated with the HOMA1-IR = fasting insulin (mU/L) x fasting glycemia (mmol/L)/22.5 equation^(^ 19 ^)^. The cutoff point for the HOMA1-IR index was set at the 75th percentile of the evaluated sample, which was stratified by sex and puberty stage, with HOMA1-IR ≥3.87 and HOMA1-IR ≥4.19 for prepubertal and pubertal females, respectively; and HOMA1-IR ≥3.85 and HOMA1-IR ≥3.77 for prepubertal and pubertal males, respectively.

Data were analyzed using the *Statistical Package for the Social Sciences* (SPSS) 16.0 software and the MedCalc 9.3.0.0 software. Significance level was set at 5% (*p*<0.05). To characterize the sample, we used descriptive statistics (mean and standard deviation or median and semi-interquartile range). The Kolmogorov-Smirnov test was used to determine the distribution of variables. To check for differences between prepubertal and pubertal males and females, we used either Student's t test or the Mann-Whitney test. The correlations between two variables were evaluated by the partial correlation coefficient adjusted for BF% and puberty stage. Qualitatively, correlations were classified as: 0-0.3 - weak; 0.3-0.6 - moderate; 0.6-0.9 - strong; and 0.9-1 - very strong. ROC (Receiver Operating Characteristic) curves were constructed and the areas under the curve (AUC) were calculated with a 95% confidence interval (IC). The sensitivity and specificity of NC and its positive and negative predictive values were calculated for each cutoff point in the sample.

This study was approved by the Unicamp Research Ethics Committee, under decision n. 900/2010, and the children's parents or guardians signed an informed consent form.

## Results

Of the 388 adolescents, 56.4% (n=219) were female. Regarding nutritional status, 51.9% of prepubertal females (n=50) had normal weight, 3.7% were overweight, and 44.4% were obese. Among pubertal females (n=169), 49.4% had normal weight, 22.1% were overweight, and 28.5% were obese. Of the 59 prepubertal males, 41.5% had normal weight, 13.8% were overweight, and 44.6% were obese, while 46.6% of pubertal males (n=110) had normal weight, 19.5% were overweight, and 33.9% were obese.

The adolescents' characteristics regarding age, anthropometry, body composition, blood pressure, and biochemistry are shown in Table 1. In females, mean NC and median systolic blood pressure (SBP) were significantly higher in pubertals than in prepubertals. Mean HbA1C and median AST and ALT were also different, with higher values for prepubertal females. The other variables did not differ between the groups. In males, mean NC and plasma uric acid, as well as median SBP and diastolic blood pressure (DBP), differed between prepubertals and pubertals, with higher values for the latter group. Mean BF%, glycemia, TC, LDL, and median HbA1C and AST were higher in prepubertal males, with a statistically significant difference. The other variables did not differ between the groups.

**Table 1:**
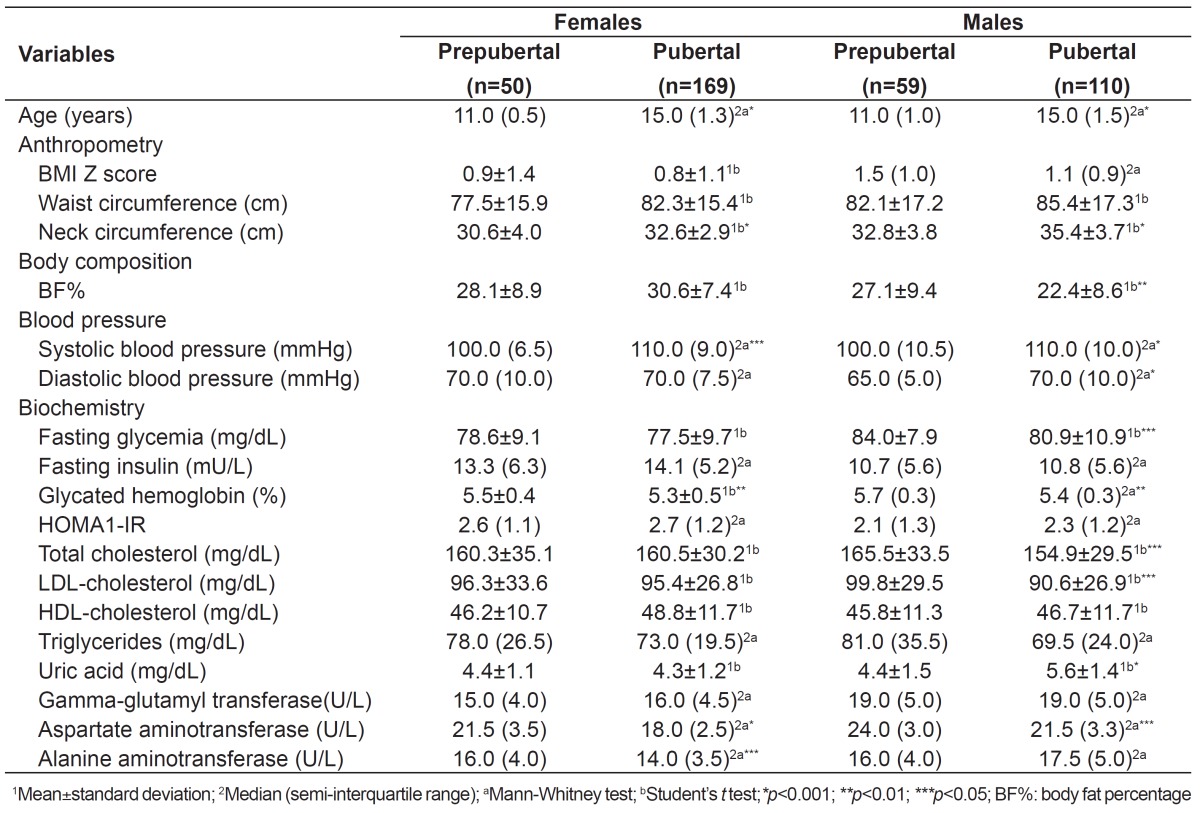
Adolescents' profiles according to age, anthropometry, body composition, blood pressure, and biochemistry

Correlations between NC, IR, and components of metabolic syndrome (SM) are presented in Table 2. NC showed a good correlation with obesity markers BMIz and WC in both sexes and puberty stages. Regarding markers of metabolic syndrome, NC showed a positive correlation with SBP, DBP, TG, plasma uric acid, GGT, and ALT and a negative correlation with HDL-cholesterol in pubertal females. A positive linear correlation was found between NC, SBP, DBP, plasma uric acid, and GGT in prepubertal males, while NC showed a positive linear correlation with SBP, DBP, LDL, TG, plasma uric acid, and GGT and a negative correlation with HDL in pubertal males. Regarding markers of IR, NC was positively correlated with insulin and HOMA1-IR in pubertal and prepubertal females. In males, there was a positive correlation only in the pubertal group. After being adjusted for BF% and puberty stage, NC showed a positive correlation with BMIz, WC, SBP, DBP, insulin, HOMA1-IR, TG, plasma uric acid, and GGT in both sexes, as well as a negative correlation with HDL. In males, NC was negatively correlated with HbA1C. In females, NC was correlated with ALT.

**Table 2:**
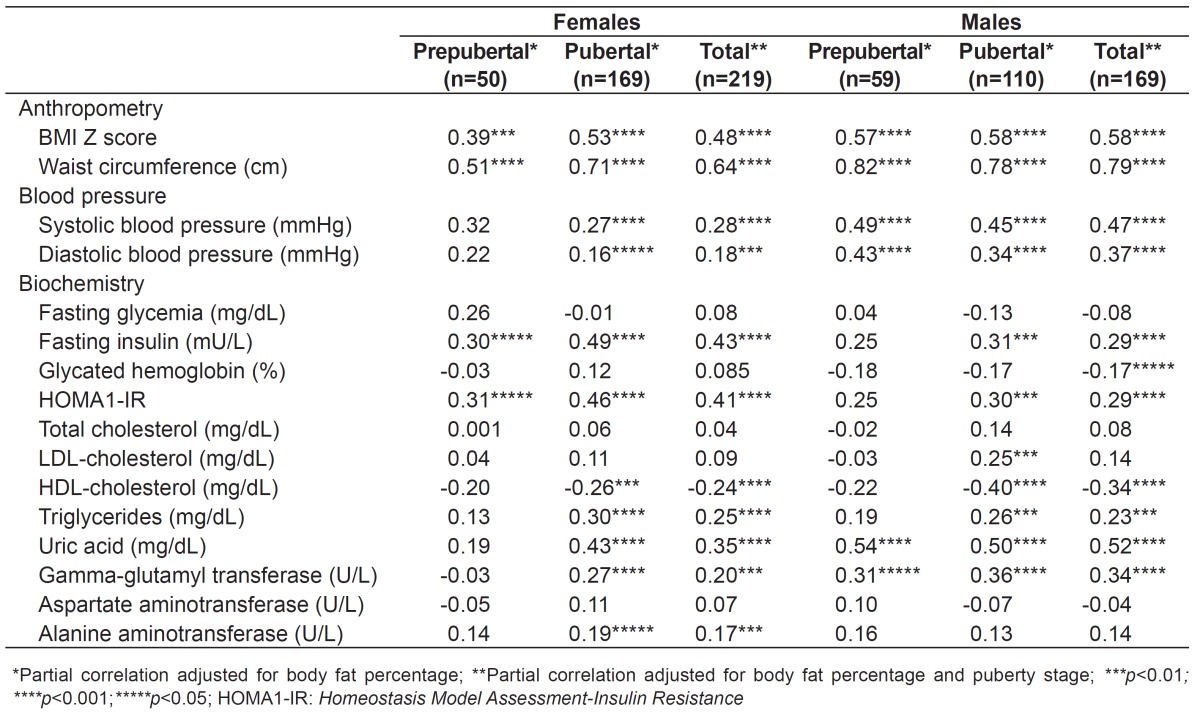
Correlation between neck circumference (in cm), insulin resistance, and components of metabolic syndrome

**Figure 1:**
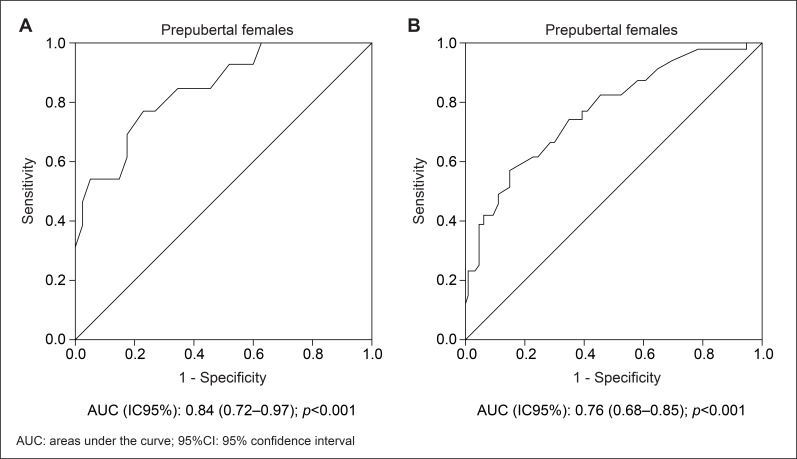
ROC curve for neck circumference in the assessment of insulin resistance in female prepubertal (A) and pubertal (B) adolescents

**Figure 2:**
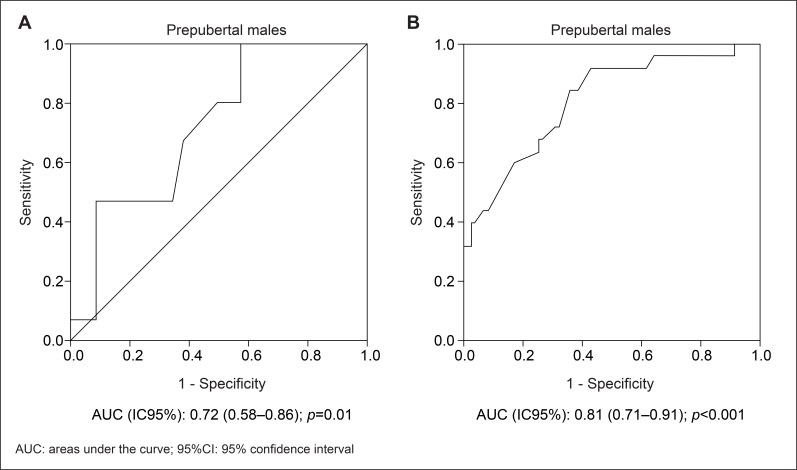
ROC curve for neck circumference in the assessment of insulin resistance in male prepubertal (A) and pubertal (B) adolescents

The areas under the ROC curve for NC as a predictor of IR in male and female pubertal and prepubertal adolescents can be seen in Figures 1 and 2, respectively. The AUCs were statistically significant (*p*<0.05). NC showed a bigger AUC for IR in prepubertal females (Figure 1A) when compared to the pubertal ones (Figure 1B). In males, NC showed a bigger AUC for IR in the pubertal group (Figure 2B) than in the prepubertal group (Figure 2A). The different cutoff points for NC and their respective sensitivities, specificities, and positive and negative predictive values are shown in Table 3. To identify IR in pubertal and prepubertal females, the cutoff points for NC were >34.1cm and >32.0cm, respectively. In males, the values were >30.3cm for prepubertal and >34.8cm for pubertal adolescents.

**Table 3:**
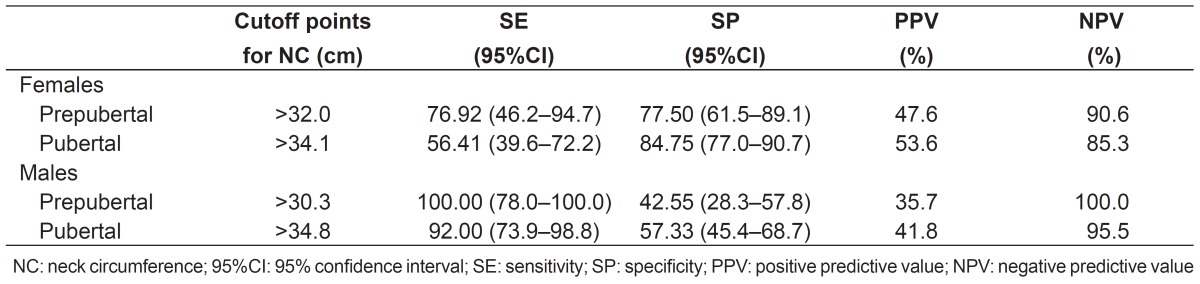
Cutoff points, sensitivities, specificities, and positive and negative predictive values of neck circumference for IR screening in adolescents

## Discussion

NC may be considered during nutritional assessment and is suggested as a substitute marker of obesity in children and adolescents in various studies^(^ 20 ^-^ 22 ^)^. This article demonstrated a significant correlation between CP and markers of obesity (BMIz and WC) in pubertal and prepubertal adolescents of both sexes. The results remained significant after the adjustment for BF% and puberty stage, which suggests NC is also a good indicator of excess body fat in Brazilian adolescents.

Guo et al^(^ 23 ^)^ and Kurtoglu et al^(^ 24 ^)^ observed a correlation between NC and anthropometric indicators of obesity. Guo et al^(^ 23 ^)^ evaluated 6,802 Chinese children and adolescents between five and 18 years old, dividing them into BMI categories, and found a significant correlation between NC and WC. Despite a decrease in correlation coefficients after the adjustment for age, sex, and BMI, NC remained positively correlated with WC. The authors also observed an association between NC and BMI in the three BMI categories, but no significance was found in the obese group after the adjustment for age, sex and WC. In the study by Kurtoglu et al^(^ 24 ^)^, with 581 Turkish children and adolescents between five and 18 years old, there was also a significant correlation between NC, WC and BMI in pubertal and prepubertal adolescents of both sexes.

Various studies with adults demonstrated that NC is a simple screening tool to identify people with cardiometabolic alterations^(^ 8 ^-^ 10 ^)^. Few studies with children evaluated NC as an indicator of IR and of alterations in components of MS^(23-25). ^When comparing our results with the ones obtained by Kurtoglu et al^(24), who analyzed the correlation between NC and ^markers of MS according to sex and puberty stage, we found that, in our previous study, there was a positive correlation between NC, SBP, DBP, glycemia, and TC in prepubertal females, and a negative correlation with HDL. This was not observed in this study, possibly because our analyses were performed using the partial correlation coefficient adjusted for BF%. Still regarding females, our article shows a positive correlation between NC, SBP, DBP, and TG, and a negative correlation between NC and HDL-cholesterol in the pubertal group, which is in agreement with the previous study. Kurtoglu et al^(^ 24 ^)^ demonstrated a positive correlation between NC and SBP, DBP, glycemia, TC, and TG in prepubertal males, while we only found a positive linear correlation with SBP and DBP. Both in the present and in the previous study, NC was positively correlated with SBP, DBP, TG, and LDL, but showed a negative linear correlation with HDL.

Androutsos et al^(^ 25 ^)^ evaluated 324 Greek children and adolescents between nine and 13 years old and detected a positive correlation between NC and DBP and a negative one with HDL in both sexes. In the same study, NC was positively correlated with DBP and TG in females. In the multivariate regression analysis after adjustment for age, sex, Tanner stage, physical activity, and intake of proteins, carbohydrates, and fat, NC had a significant positive correlation with HDL, TG, SBP and DBP. The authors also noticed that the associations between NC and the risk factors for CVD were similar ​​to the ones observed with BMIz, WC, hip circumference, waist-to-hip ratio and waist-to-height ratio in children and adolescents. In our study, after being adjusted for BF% and puberty stage, NC showed a positive correlation with SBP, DBP, and TG in both sexes, as well as a negative correlation with HDL.

The Chinese study evaluated the correlation between NC and blood pressure in different BMI groups. In normal weight subjects, the biggest NC was associated with an increased risk of pre-hypertension (OR 1.64, 95%CI 1.29-2.08) after adjustments for age and sex. This result remained significant after adjustments for age, sex, BMI, and WC (OR 1.44; 95%CI 1.12-1.85). In the overweight and obese groups, no significant odds ratios were observed. In the normal weight group, there was an association between NC, SBP (β=0.58mmHg), and DBP (β=0.24mmHg)^(^ 23 ^)^.

Regarding markers of IR, Kurtoglu et al^(^ 24 ^)^ found a positive correlation between NC, insulin, and HOMA1-IR in pubertal and prepubertal adolescents of both sexes. Our study, however, demonstrated a correlation both in pubertal and prepubertal females, but only in pubertal males. Androutsos et al^(^ 25 ^)^ presented a positive correlation of NC with insulin and HOMA-IR in both sexes. The results remained significant after adjustments for age, sex, Tanner stage, physical activity, and intake of proteins, carbohydrates and fat. In our study, after being adjusted for BF% and puberty stage, NC showed a positive correlation with insulin and HOMA1-IR in males and females.

We also evaluated the association between NC, plasma uric acid, GGT, ALT, and AST in adolescents, which were previously analyzed in adults only. In a study we conducted with adults, NC was associated with plasma uric acid and GGT only in women^(^ 8 ^)^. In the current investigation, NC was correlated with plasma uric acid, GGT, and ALT in pubertal females, while it was correlated with plasma uric acid and GGT both in prepubertal and pubertal males. After the adjustment for BF% and puberty stage, NC was correlated with plasma uric acid and GGT in both sexes and with ALT in females.

Our study is the first to determine the usefulness of NC as a parameter in the prediction of IR in adolescents by using ROC curves. The results showed that NC is a good predictor of IR in adolescents. The mechanisms involved in the association between NC and cardiometabolic risk factors were not defined. Data in the literature indicate that subcutaneous fat in the upper body, as well as visceral fat, is associated with cardiometabolic risks^(^ 10 ^)^. This is possibly due to the fact that visceral fat is not the main source of concentrations of circulating free fatty acid (FFA)^(^ 26 ^)^. Besides, subcutaneous fat in the upper body causes an increase in systemic free fatty acid release when compared to visceral fat, particularly in obese individuals^(^ 27 ^)^. Similarly, high concentrations of FFA were associated with IR and a higher cardiovascular risk^(^ 28 ^,^ 29 ^)^. Since this upper-body subcutaneous fat can be easily assessed by NC, this measurement may be an important predictor of IR and of cardiometabolic risks. It may also contribute to a better understanding of the effects of body fat distribution in adolescents. Further longitudinal studies are needed to analyze the relationship between NC, IR, and alterations in components of MS in adolescents.

Our results show that NC has good sensitivity for the identification of IR and could even be used as a screening method. When comparing this study with our previous study with adults, which also suggested NC is a useful tool for predicting IR, we concluded that the cutoff point for adolescents is lower^(^ 8 ^)^.

NC has some advantages over WC: good inter-rater and intra-rater reliability; no need for multiple accuracy and reliability measurements^(^ 30 ^)^; no influence of time of measurement (preprandial and postprandial period); more stable body surface; easier for both examiners and participants, particularly during winter and in crowded locations; more socially acceptable and more convenient, especially for overweight and obese adolescents. However, NC does not have international reference values yet.

The present study has the following limitations: 1) the results are based on a cross-sectional study, which precludes the identification of causality; 2) the sample was selected by convenience, with a higher proportion of obese individuals; 3) we did not analyze the correlations with imaging studies that directly quantify fat deposits. Despite these limitations, our results were in agreement with the results of the cited studies and showed that NC may be an important health indicator in adolescents, since it is a screening tool that can identify IR and alterations in the components of MS in Brazilian adolescents. Its simplicity and low cost may enable its use in Public Health services and in epidemiological studies.

## Investigadores do Brams (Brazilian Metabolic Syndrome Study):

Ana Carolina Junqueira Vasques, Ana Maria De Bernardi Rodrigues, André Luiz Gonçalves de Freitas, Bruno Geloneze, Cleliani de Cassia da Silva, Daniella Fernandes Camilo, Fabiana Lopes Nogueira, Francieli Barreiro, José Carlos Pareja, Maria Ângela Reis de Góes Monteiro Antonio, Mariana Pontes Ferrari, Mariana Porto Zambom, Patrícia Brito Rodrigues, Roberta Soares Lara Cassani.
