# Supplementary material for: Neck circumference as a new anthropometric indicator for prediction of insulin resistance and components of metabolic syndrome in adolescents: Brazilian Metabolic Syndrome Study
Source: Rev Paul Pediatr. 2014 Jun;32(2):221–9. doi: 10.1590/0103-0582201432210713 (PMC4183009; doi:10.1590/0103-0582201432210713)
Supplement: Translation

# Translation: Circunferência do pescoço como um novo indicador antropométrico para predição de resistência à insulina e componentes da síndrome metabólica em adolescentes: *Brazilian Metabolic Syndrome Study*

**Authors:** Cleliani de Cassia da Silva, Mariana Porto Zambon, Ana Carolina J. Vasques, Ana Maria de B. Rodrigues, Daniella Fernandes Camilo, Maria Ângela R. de G. M. Antonio, Roberta Soares L. Cassani, Bruno Geloneze

**Affiliations:** 1 Faculdade de Ciências Médicas da Unicamp; Laboratório de Investigação em Metabolismo e Diabetes (Limed) - Gatrocentro-Unicamp, Campinas, SP, Brasil

**Keywords:** resistência à insulina, síndrome x metabólica, adolescente, adiposidade

## Abstract

**OBJETIVO:**

Avaliar a correlação da circunferência do pescoço com resistência à insulina e com os componentes da síndrome metabólica em adolescentes com diferentes níveis de adiposidade e estadios puberais, bem como determinar a utilidade da circunferência do pescoço como um parâmetro na predição de resistência à insulina em adolescentes.

**MÉTODOS:**

Estudo transversal no qual se avaliaram 388 adolescentes de ambos os sexos, de dez a 19 anos. Os adolescentes foram submetidos à avaliação antropométrica e de composição corporal, incluindo circunferências do pescoço e da cintura, e a avaliação bioquímica. O estadio puberal foi obtido por meio de autoavaliação e a pressão arterial, pelo método auscultatório. Analisou-se a resistência à insulina pelo *Homeostasis Model Assessment-Insulin Resistance*. A correlação entre duas variáveis foi verificada com o coeficiente de correlação parcial ajustado para o percentual de gordura corporal e o estadio puberal. O desempenho da circunferência do pescoço para identificar resistência à insulina foi testado pela *Receiver Operating Characteristic Curve*.

**RESULTADOS:**

Após ajuste para o percentual de gordura corporal e estadio puberal, a circunferência do pescoço correlacionou-se com circunferência da cintura, pressão arterial, triglicérides e marcadores de resistência à insulina em ambos os sexos.

**CONCLUSÕES:**

Os resultados demonstraram que a circunferência do pescoço é uma ferramenta útil para detectar a resistência à insulina e a alteração nos indicadores de síndrome metabólica em adolescentes. A facilidade de aplicação e o baixo custo podem viabilizar sua utilização em serviços de Saúde Pública.

## Introdução

A composição corporal e a distribuição da gordura corporal relacionam-se a complicações como resistência à insulina (RI), dislipidemia, diabetes melito tipo 2 e doenças cardiovasculares (DCV), tanto em adultos quanto em crianças e adolescentes^(^ ^1^ ^,^ ^2^ ^)^. Os exames de imagem - tomografia computadorizada, ressonância magnética e absortometria radiológica de dupla energia (DEXA) - são considerados padrão-ouro como ferramentas para avaliar a adiposidade corporal, porém sua realização é limitada e de alto custo. Em estudos epidemiológicos e na prática clínica, valorizam-se medidas antropométricas - índice de massa corpórea (IMC), circunferência da cintura (CC) e do pescoço (CP) - por serem medidas mais acessíveis, rápidas, não invasivas, com menor custo e, portanto, com maior facilidade de aplicação^(^ ^3^ ^)^.

Atualmente, com referenciais internacionais padronizados, o IMC é a medida mais utilizada para o diagnóstico de sobrepeso e obesidade em crianças e adolescentes, porém nem sempre é capaz de avaliar o risco de complicações endócrinas e metabólicas em nível individual, pois não permite avaliar a distribuição de gordura corporal^(^ ^3^ ^)^. A medida da CC tem sido utilizada no diagnóstico de síndrome metabólica (SM), para predizer a RI e na avaliação de fatores de risco para DCV em adolescentes^(^ ^4^ ^)^, porém existem limitações nessa faixa etária, como ausência de metodologia padrão para aferição^(5-7),^ falta de padrão internacional devido à variação étnica, ausência de ponto de corte para risco cardiovascular e metabólico, dificuldades práticas de aferição, especialmente no inverno, devido à necessidade de remoção de roupas, com ênfase psicológica nesse grupo etário^(^ ^8^ ^)^. 

Como alternativa à CC, estudos em adultos têm sugerido a utilização da CP como indicador antropométrico mais simples, prático, não influenciado pela distensão abdominal pós-prandial ou por movimentos respiratórios e que fornece resultados consistentes para indicar o acúmulo de gordura subcutânea da parte superior do corpo^(^ ^8^ ^-^ ^10^ ^)^. O aumento da CP associa-se a riscos cardiometabólicos, tanto quanto a gordura visceral abdominal^(^ ^10^ ^)^. Também em adultos, demonstrou-se correlação positiva da CP com a RI, com os componentes da SM e com os fatores de riscos cardiovasculares^(^ ^8^ ^-^ ^10^ ^)^. Em adolescentes, os estudos que avaliam a CP são escassos. 

Portanto, considerando-se a facilidade de obtenção da CP em estudos epidemiológicos e na prática clínica e a identificação precoce de alterações metabólicas que contribui para prevenir doenças, o objetivo do estudo foi: 1) avaliar a correlação entre a CP com a RI e com os componentes da SM em adolescentes com diferentes níveis de adiposidade e estadios puberais e 2) identificar se a CP pode ser um preditor de RI nessa faixa etária. 

## Método

Estudo transversal, parte de uma investigação de maior abrangência intitulada *Brazilian Metabolic Syndrome Study *(Brams), que avalia aspectos clínicos, antropométricos, metabólicos e hormonais da síndrome de resistência à insulina em crianças e adolescentes. 

Avaliaram-se 388 adolescentes de ambos os sexos, de dez a 19 anos. Selecionou-se a amostra por conveniência, sendo constituída por adolescentes dos ambulatórios de obesidade na criança e no adolescente do Hospital de Clínicas da Universidade Estadual de Campinas (Unicamp), das Unidades Básicas de Saúde, de escolas públicas e de instituições que oferecem programas socioeducativos para adolescentes. Incluíram-se no estudo adolescentes eutróficos, com sobrepeso e obesidade, segundo os critérios do *Centers for Disease Control and Prevention* (CDC)^(^ ^11^ ^)^. Consideraram-se critérios de exclusão: presença de massas cervicais ou deformidades no pescoço, bócio, atraso no desenvolvimento neuropsicomotor, síndromes genéticas, hepatopatia, nefropatia, distúrbios metabólicos (como diabetes tipo 1, hipotiroidismo e hipertiroidismo) e uso de corticoide sistêmico.

Avaliaram-se o peso corporal e a estatura segundo as técnicas propostas por Gordon *et al**^(^* ^12^ ^)^ e, a partir dessas medidas, determinou-se o escore Z do índice de massa corpórea (zIMC) para idade. Aferiu-se a CC com fita métrica no ponto médio entre a crista ilíaca e a última costela^(^ ^13^ ^)^. A CP foi aferida na altura média do pescoço^(^ ^9^ ^)^. Avaliou-se o percentual de gordura corporal (%GC) pelo método de bioimpedância tetrapolar^(^ ^14^ ^,^ ^15^ ^)^, com o equipamento *Bioimpedance Analyzer* - modelo BIA 310^(r)^, segundo as orientações propostas no protocolo de Lukaski *et al**^(^* ^14^ ^)^. Avaliou-se a pressão arterial pela técnica auscultatória com esfigmomanômetro de coluna de mercúrio, de acordo com os procedimentos preconizados pela Sociedade Brasileira de Cardiologia^(^ ^16^ ^)^. A maturação sexual foi definida por autoavaliação, segundo os critérios propostos por Tanner^(^ ^17^ ^)^. Para a classificação em pré-púberes (1-2) e púberes (3-5), consideraram-se as mamas no sexo feminino e a genitália externa no masculino.

Para avaliação bioquímica, coletaram-se amostras de sangue venoso após jejum noturno de 12 horas. As determinações plasmáticas de glicose, colesterol total (CT), HDL-colesterol (HDL), triglicérides (TG) e gama glutamil transferase (GGT) foram realizadas pelo método colorimétrico enzimático e a fração LDL-colesterol (LDL) foi calculada segundo a equação de Friedewald *et al**^(^* ^18^ ^)^. Mediu-se o *ácido úrico *plasmático (URAC) pelo método uricase, a hemoglobina glicada (HbA1C) por cromotografia líquida de alta performance (HPLC), as concentrações séricas de aspartato-aminotransferase (AST) e alanina-aminotranferase (ALT) pelo método cinético UV e a insulina plasmática por quimioluminescência. Avaliou-se a RI pelo índice *Homeostasis Model Assessment-Insulin Resistance* (HOMA1-IR), calculado a partir da equação HOMA1-IR = insulina de jejum (mU/L) x glicemia de jejum (mmol/L)/22,5^(^ ^19^ ^)^. O ponto de corte utilizado do índice HOMA1-IR foi o percentil 75 da amostra avaliada, estratificado por sexo e estadio puberal, sendo HOMA1-IR ≥3,87 e HOMA1-IR ≥4,19 para o sexo feminino pré-púbere e púbere respectivamente; para o sexo masculino pré-púbere, utilizou-se HOMA1-IR ≥3,85 e, para os púberes, HOMA1-IR ≥3,77.

Analisaram-se os dados nos *softwares Statistical Package for the Social Sciences* (SPSS), versão 16.0, e MedCalc, versão 9.3.0.0. O nível de significância adotado foi de 5% (*p*<0,05). Para caracterizar a amostra, utilizou-se a estatística descritiva (média e desvio padrão ou mediana e semiamplitude interquartílica). Aplicou-se o teste de Kolmogorov-Smirnov para verificar a distribuição das variáveis; para comparar as diferenças entre os sexos e entre pré-púberes *versus* púberes, aplicou-se o teste *t* de Student ou o teste de Mann-Whitney. As correlações entre duas variáveis foram avaliadas pelo coeficiente de correlação parcial ajustado para %GC e estadio puberal. Definiu-se a intensidade qualitativa da correlação como: 0-0,3 - fraca; 0,3-0,6 - regular; 0,6-0,9 - forte e 0,9-1 - muito forte. Construíram-se curvas ROC (*Receiver Operating Characteristic Curve*) e calcularam-se as áreas abaixo das curvas (AUC), com intervalo de confiança (IC) de 95%. A sensibilidade e a especificidade da CP e seus valores preditivos positivos e negativos foram calculados para cada ponto de corte na amostra.

O estudo foi aprovado pelo Comitê de Ética em Pesquisa da Unicamp e os pais ou responsáveis assinaram o termo de consentimento livre e esclarecido.

## Resultados

Do total de 388 adolescentes, 56,4% (n=219) eram do sexo feminino. Quanto ao estado nutricional, das 50 meninas pré-púberes avaliadas, 51,9% eram eutróficas, 3,7% apresentaram sobrepeso e 44,4%, obesidade, enquanto que, das 169 meninas púberes avaliadas, 49,4% eram eutróficas, 22,1% estavam com sobrepeso e 28,5%, com obesidade. Quanto ao sexo masculino, dos 59 meninos pré-púberes avaliados, 41,5% eram eutróficos, 13,8% estavam com sobrepeso e 44,6% com obesidade, enquanto que, dos 110 púberes avaliados, 46,6% eram eutróficos, 19,5% estavam com sobrepeso e 33,9%, com obesidade. 

As características dos adolescentes segundo idade, antropometria, composição corporal, pressão arterial e bioquímica estão apresentadas na Tabela 1. No sexo feminino, as médias de CP e as medianas da pressão arterial sistólica (PAS) diferiram significativamente entre pré-púberes e púberes, sendo mais elevadas nas púberes. As médias de HbA1C e as medianas de AST e ALT também diferiram entre as pré-púberes e púberes, sendo mais elevada nas pré-púberes. As demais variáveis não diferiram entre as meninas pré-púberes e púberes. No sexo masculino, as médias de CP e de URAC, bem como as medianas de PAS e de pressão arterial diastólica (PAD) diferiram entre pré-púberes e púberes, sendo mais elevadas nos púberes, ao passo que as médias de %GC, glicemia, CT, LDL e as medianas de HbA1C e AST foram mais elevadas nos pré-púberes, com diferença estatisticamente significante. As demais variáveis não diferiram entre meninos pré-púberes e púberes.

**Tabela 1:** Caracterização dos adolescentes segundo idade, antropometria, composição corporal, pressão arterial e bioquímica 1Média±desvio

As correlações entre a CP, a RI e os componentes da síndrome metabólica (SM) são apresentadas na Tabela 2. A CP apresentou correlação com os marcadores de obesidade, zIMC e CC em ambos os sexos, em pré-púberes e púberes. Entre os marcadores da SM, no sexo feminino, a CP mostrou correlação positiva com PAS, PAD, TG, URAC, GGT e ALT, sendo a correlação negativa com HDL-colesterol nas púberes. No sexo masculino, houve correlação linear positiva entre CP, PAS, PAD, URAC e GGT nos pré-púberes, ao passo que, nos púberes, a CP apresentou correlação linear positiva com PAS, PAD, LDL, TG, URAC e GGT e correlação negativa com HDL. Quanto aos marcadores de RI no sexo feminino, a CP apresentou correlação positiva com insulina e HOMA1-IR nas pré-púberes e púberes; já no sexo masculino, houve correlação positiva apenas nos púberes. Após ajuste para o %GC e estadio puberal, a CP correlacionou-se de forma positiva com zIMC, CC, PAS, PAD, insulina, HOMA1-IR, TG, URAC e GGT e de, forma negativa, com HDL, em ambos os sexos. No sexo masculino, a CP correlacionou-se negativamente com HbA1C. No sexo feminino, a CP correlacionou-se com ALT.

**Tabela 2:** Correlação entre a circunferência do pescoço (em cm), resistência à insulina e componentes da síndrome metabólica

As áreas sob a curva ROC para CP como preditora de RI em adolescentes do sexo feminino e masculino, pré-púberes e púberes, podem ser observadas nas Figuras 1 e 2, respectivamente. As AUC foram estatisticamente significantes (*p*<0,05). No sexo feminino, a CP apresentou a maior AUC para a RI nas pré-púberes (Figura 1A) em relação às púberes (Figura 1B). Já no sexo masculino, a CP apresentou maior AUC para a RI nos púberes (Figura 2B) em relação aos pré-púberes (Figura 2A). Os diferentes pontos de corte da CP e as respectivas sensibilidades e especificidades e seus valores preditivos positivos e negativos estão apresentados na Tabela 3. Encontraram-se os seguintes pontos de corte da CP para identificar a RI no sexo feminino pré-púbere e púbere: >32,0cm e >34,1cm, respectivamente. Para o sexo masculino, os valores foram >30,3cm para os pré-púberes e >34,8cm para os púberes.

**Figura 1:** Curva ROC para a circunferência do pescoço na avaliação da resistência à insulina em adolescentes do sexo feminino pré-púberes (A) e púberes (B)

**Figura 2:** Curva ROC para a circunferência do pescoço na avaliação da resistência à insulina em adolescentes do sexo masculino pré-púberes (A) e púberes (B)

**Tabela 3:** Pontos de corte, sensibilidades, especificidades e valores preditivos positivos e negativos da circunferência do pescoço para rastreamento de resistência à insulina em adolescentes

## Discussão

A CP pode ser acrescida na avaliação nutricional, sendo proposta como marcador substituto de obesidade em crianças e adolescentes em vários estudos^(^ ^20^ ^-^ ^22^ ^)^. O presente trabalho demonstrou correlação significante entre a CP e os marcadores de obesidade (zIMC e CC) nos adolescentes pré-púberes e púberes de ambos os sexos. Os resultados permaneceram significativos após ajuste para o %GC e estadio puberal, indicando que a CP também é um bom indicador de acúmulo de gordura corporal em adolescentes brasileiros. 

Guo *et al**^(^* ^23^ ^)^ e Kurtoglu *et al**^(24) ^*observaram correlação entre CP e indicadores antropométricos de obesidade. Guo *et al**^(^* ^23^ ^)^ avaliaram 6.802 crianças e adolescentes chineses de cinco a 18 anos divididos por categoria de IMC e observaram correlação significante entre CP e CC. Apesar da redução dos coeficientes de correlação após ajuste para idade, sexo e IMC, a CP manteve correlação positiva com CC. Os autores também observaram associação entre CP e IMC em todas as três categorias de IMC, mas não observaram significância no grupo de obesos após ajuste para idade, sexo e CC. No estudo de Kurtoglu *et al**^(^* ^24^ ^)^ com 581 crianças e adolescentes turcos de cinco a 18 anos, também houve correlação significante entre CP, CC e IMC em adolescentes pré-púberes e púberes de ambos os sexos.

Vários estudos com adultos documentaram que a CP é uma ferramenta simples de triagem para identificar indivíduos com alterações cardiometabólicas^(^ ^8^ ^-^ ^10^ ^)^. Poucos estudos pediátricos exploraram a CP como indicador de RI e de alterações nos componentes da SM^(23-25). Comparando nossos resultados com os de ^Kurtoglu *et al**^(24), que avaliaram a correlação da CP com marcadores da SM conforme sexo e estadio puberal, verificou-se que, no estudo anterior, nas pré-púberes d^*o sexo feminino, houve correlação positiva entre CP, PAS, PAD, glicemia e CT e correlação negativa com HDL, o que não se observou neste estudo, possivelmente pelo fato de nossas análises usarem o coeficiente de correlação parcial ajustado para %GC. Ainda quanto ao sexo feminino, o presente trabalho demonstra que a CP correlacionou-se positivamente com a PAS, PAD e TG e negativamente com HDL-colesterol nas púberes, semelhantemente ao estudo anterior. No sexo masculino, no estudo de Kurtoglu *et al**^(^* ^24^ ^)^, a CP correlacionou-se positivamente com PAS, PAD, glicemia, CT e TG nos pré-púberes . Neste estudo, houve correlação linear positiva apenas com PAS e PAD. Quanto aos púberes, em nosso estudo, assim como no anterior, a CP apresentou correlação linear positiva com PAS, PAD, TG e LDL e correlação linear negativa com HDL. 

Androutsos *et al**^(^* ^25^ ^)^ avaliaram 324 crianças e adolescentes gregos de nove a 13 anos, tendo encontrado correlação positiva entre CP e PAS e negativa com HDL, em ambos os sexos. Nesse mesmo estudo, a CP apresentou correlação positiva com PAD e TG no sexo feminino. Na análise de regressão multivariada após ajuste para idade, sexo, estadio de Tanner, atividade física, ingestão dietética de proteínas, carboidratos e gordura, a CP correlacionou-se significativamente com HDL, TG, PAS e PAD. Os autores também identificaram que as associações entre a CP e os fatores de risco para DCV eram comparáveis ​​às notadas para zIMC, CC, circunferência do quadril, relação cintura-quadril e relação cintura-altura em crianças e adolescentes. Em nosso estudo, após ajuste para o %GC e estadio puberal, a CP correlacionou-se de forma positiva com PAS, PAD e TG e de forma negativa com HDL, em ambos os sexos. 

A pesquisa de crianças e adolescentes chineses avaliou a correlação entre CP e pressão arterial em diferentes grupos de IMC. Nos participantes com peso normal, a CP mais elevada associou-se a maior risco de pré-hipertensão (OR 1,64, IC95% 1,29-2,08), após ajustes para idade e sexo. Esse resultado permaneceu significativo após ajustes para idade, sexo, IMC e CC (OR 1,44; IC95% 1,12-1,85). Nas categorias de sobrepeso e obesidade, não se observaram razões de chance significantes. No grupo de peso normal, houve associação entre CP, PAS (β=0,58mmHg) e PAD (β=0,24mmHg)^(^ ^23^ ^)^.

Quanto aos marcadores de RI, no estudo de Kurtoglu *et al**^(^* ^24^ ^)^, a CP correlacionou-se positivamente com insulina e HOMA1-IR em ambos os sexos, em pré-púberes e púberes. Já no presente estudo, houve correlação no sexo feminino nas pré-púberes e púberes, enquanto, no masculino, apenas nos púberes. No estudo de Androutsos *et al**^(^* ^25^ ^)^, a CP apresentou correlação positiva com a insulina e o HOMA-IR em ambos os sexos. Os resultados permaneceram significativos após ajustes para idade, sexo, estadio de Tanner, atividade física, ingestão dietética de proteínas, carboidratos e gordura. No nosso estudo, após ajuste para %GC e estadio puberal, a CP correlacionou-se com insulina e HOMA1-IR em ambos os sexos.

O presente trabalho também avaliou a associação entre CP, URAC, GGT, ALT e ALT em adolescentes, o que havia sido pesquisado anteriormente apenas em adultos. Em estudo do nosso grupo realizado com adultos, a CP apresentou associação com URAC e GGT apenas nas mulheres^(^ ^8^ ^)^. Na investigação atual, a CP correlacionou-se com URAC, GGT e ALT nas meninas púberes, enquanto que, no sexo masculino, a CP correlacionou-se com URAC e GGT nos pré-púberes e com URAC e GGT nos púberes. Após ajuste para %GC e estadio puberal, a CP correlacionou-se com URAC e GGT em ambos os sexos e com ALT no sexo feminino.

Nosso estudo é pioneiro em determinar a utilidade da CP como parâmetro na predição de RI em adolescentes por meio de curvas ROC. Os resultados mostraram que a CP é um bom preditor de RI em adolescentes. Ainda não se definiram os mecanismos envolvidos para justificar a associação entre a CP e os fatores de risco cardiometabólicos. Dados de literatura indicam que a gordura subcutânea da parte superior do corpo relaciona-se aos riscos cardiometabólicos, tanto quanto a gordura visceral abdominal^(^ ^10^ ^)^, possivelmente pelo fato de a gordura visceral não ser a principal fonte de concentrações circulantes de ácidos graxos livres (AGL)^(^ ^26^ ^)^. Além disso, a gordura subcutânea da parte superior do corpo é responsável por uma maior liberação sistêmica de AGL do que a gordura visceral, particularmente em indivíduos obesos^(^ ^27^ ^)^. Da mesma forma, elevadas concentrações de AGL têm sido associadas à RI e ao aumento do risco cardiovascular^(^ ^28^ ^,^ ^29^ ^)^. Como essa gordura subcutânea da parte superior do corpo é facilmente avaliada pela CP, esta pode ser um importante preditor de RI e de risco cardiometabólico, além de contribuir para uma melhor compreensão dos efeitos da distribuição de gordura corporal em adolescentes. Estudos longitudinais são necessários para examinar a relação entre CP, RI e alterações nos componentes da SM em adolescentes.

Nossos resultados mostram que a CP tem boa sensibilidade para identificar a RI, podendo ser inclusive utilizada como método de triagem. Comparando tais resultados com os obtidos em adultos, que também demonstraram ser a CP útil para predição de RI, verificou-se que o ponto de corte em adolescntes é menor^(^ ^8^ ^)^.

Comparada à medida da CC, a aferição da CP apresenta algumas vantagens: boa confiabilidade inter e intraobservador; não requer múltiplas medições de precisão e confiabilidade^(^ ^30^ ^)^; não é influenciada pelo horário de avaliação (período pré-prandial e pós-prandial); é aferida em superfície do corpo mais estável; apresenta maior facilidade tanto para o examinador quanto para o examinando, especialmente no inverno e em locais movimentados; é mais aceitável socialmente e conveniente, principalmente para adolescentes com sobrepeso e obesidade. No entanto, ainda não apresenta valores internacionais de referência.

O presente estudo apresenta limitações: 1) os resultados são baseados em estudo transversal, o que não permite inferir causalidade; 2) selecionou-se a amostra por conveniência, com maior proporção de obesos; 3) não foram realizadas correlações com exames de imagem que quantificam diretamente o depósito de gordura. Apesar das limitações, de forma consistente com os estudos citados, os resultados desta investigação mostraram que a CP pode ser um importante indicador da saúde de adolescentes, por ser um instrumento de rastreamento capaz de identificar a RI e as alterações nos componentes da SM em adolescentes brasileiros. A facilidade de aplicação e o baixo custo podem viabilizar sua utilização em serviços de Saúde Pública e em estudos epidemiológicos. 

## Investigadores do Brams (Brazilian Metabolic Syndrome Study):

Ana Carolina Junqueira Vasques, Ana Maria De Bernardi Rodrigues, André Luiz Gonçalves de Freitas, Bruno Geloneze, Cleliani de Cassia da Silva, Daniella Fernandes Camilo, Fabiana Lopes Nogueira, Francieli Barreiro, José Carlos Pareja, Maria Ângela Reis de Góes Monteiro Antonio, Mariana Pontes Ferrari, Mariana Porto Zambom, Patrícia Brito Rodrigues, Roberta Soares Lara Cassani.
